# The impact of thyroid hormones on patients with hepatocellular carcinoma

**DOI:** 10.1371/journal.pone.0181878

**Published:** 2017-08-03

**Authors:** Matthias Pinter, Lukas Haupt, Florian Hucke, Simona Bota, Theresa Bucsics, Michael Trauner, Markus Peck-Radosavljevic, Wolfgang Sieghart

**Affiliations:** 1 Division of Gastroenterology & Hepatology, Department of Internal Medicine III, Medical University of Vienna, Vienna, Austria; 2 Liver Cancer (HCC) Study Group Vienna, Vienna, Austria; 3 Department of Gastroenterology & Hepatology, Endocrinology and Nephrology, Klinikum Klagenfurt am Wörthersee, Klagenfurt, Austria; Chang Gung Memorial Hospital Kaohsiung Branch, TAIWAN

## Abstract

**Background & aims:**

Hypothyroidism has recently been proposed as predisposing factor for HCC development. However, the role of thyroid hormones (TH) in established HCC is largely unclear. We investigated the impact of TH on clinical characteristics and prognosis of HCC patients.

**Methods:**

Of 838 patients diagnosed with nonsurgical HCC at the Division of Gastroenterology and Hepatology/Medical University of Vienna between 1992 and 2012, 667 patients fulfilled the inclusion criteria. The associations of thyroid function tests with patient, liver, and tumor characteristics as well as their impact on overall survival (OS) were investigated.

**Results:**

Thyroid hormone substitution was more often observed in patients with low thyroid-stimulating hormone (TSH) concentration and in patients with elevated free tetraiodthyronine (fT4). Patients with high TSH (>3.77uU/ml) concentrations had larger tumors, while the opposite was true for patients with low TSH (<0.44uU/ml) concentrations. Subjects with elevated fT4 (>1.66ng/dl) were more likely to have elevated CRP. While TSH was only associated with OS in univariate analysis (≤1.7 vs. >1.7uU/ml, median OS (95%CI), 12.3 (8.9–15.7 months) vs. 7.3 months (5.4–9.2 months); p = 0.003), fT_4_ (≤1.66 vs. >1.66ng/dl, median OS (95%CI), 10.6 (7.5–13.6 months) vs. 3.3 months (2.2–4.3 months); p = 0.007) remained an independent prognostic factor for OS (HR (95%CI) for fT_4_>1.66ng/dl, 2.1 (1.3–3.3); p = 0.002) in multivariate analysis.

**Conclusions:**

TSH and fT_4_ were associated with prognostic factors of HCC (i.e., tumor size, CRP level). Elevated fT_4_ concentrations were independently associated with poor prognosis in HCC. Further studies are needed to characterize the role of TH in HCC in detail.

## Introduction

Components of thyroid hormone signaling are implicated in the development and progression, as well as in the prevention of various cancers including hepatocellular carcinoma (HCC) [1–3]. HCC usually develops in patients with liver cirrhosis [4, 5] and represents the second most common cause of cancer-related mortality in men globally [6].

The thyroid hormones (TH) tetraiodthyronine (T_4_) and, to a lesser extent, triiodthyronine (T_3_) are produced in the thyroid gland in a complex multi- step process, which is tightly controlled by the hypothalamus-pituitary-thyroid axis. Hypothalamic neurons produce the tripeptide thyrotropin-releasing-hormone (TRH), which uses the pituitary portal venous system to reach the anterior pituitary gland, where it binds to the TRH receptor in thyrotropic cells, and thereby stimulates both the production and pulsatile release of the glycoprotein thyrotropin (thyroid-stimulating hormone, TSH). By binding to the TSH-receptor situated at the basolateral membrane of thyrocytes, TSH activates thyroid hormone production and secretion.

The vast majority of both thyroid hormones is bound to the plasma proteins and therefore unable to bind to receptors in target tissues. This constellation allows preparation of a large pool of hormones, which can quickly be released when needed. Conversely, the smaller, unbound fraction of thyroid hormones is responsible for the actual physiological effects. The thyroid gland predominantly secretes T_4_, most of which is subjected to peripheral metabolism by deiodinase enzymes [7].

Since TH display extensive influence on regulatory mechanisms affecting the control of cellular growth and metabolism [7–10], there are myriad possible modes of interaction with both the development and progression of HCC. Although hypothyroidism has been shown to be associated with an elevated risk of HCC development [2, 11, 12], the role of TH in established HCC remains to be elucidated. Several preclinical studies have linked TH signaling to tumor-promoting actions, especially at advanced stages of hepatocarcinogenesis [13–15]. However, little is known about both the prevalence and clinical role of TH in patients with manifest HCC. Hence, we aimed to investigate the association of TH with clinical patient and tumor characteristics as well as their impact on the prognosis of HCC patients.

## Materials and methods

### Patient selection

Data from all patients who were diagnosed with HCC by biopsy or radiological imaging according to the European Association for the Study of the Liver (EASL) [16] diagnostic criteria between August 1992 and February 2013 at the Medical University of Vienna were retrospectively collected and incorporated into a database. Patients who had received surgical treatment for HCC at any time after diagnosis were excluded from analysis. Only patients aged ≥18 years with TSH levels available at the time of diagnosis were eligible. Collection and retrospective analysis was approved by the Ethics Committee of the Medical University of Vienna.

### Data acquisition

The date of HCC diagnosis (date of biopsy if available or diagnostic imaging) was considered the baseline of this study. Patient information was collected in a preexisting MS Access 2010 database (HCC database) in a pseudo-anonymous manner. Patient characteristics, laboratory parameters including thyroidal function (TSH, T3, T4, fT3, fT4), tumor characteristics, and parameters representing liver function were recorded from the patients’ charts and from the electronic patients’ information system. Determination of thyroid status was based on TSH and fT_4_ levels. We formed 5 subgroups: euthyreosis, hyperthyreosis (primary, secondary, and euthyroid hyperthyroxinemia), subclinical hyperthyreosis, subclinical hypothyreosis, hypothyreosis (primary and secondary), and thyroid hormone substitution. Liver function was assessed by MELD score and Child-Pugh score. The latter has been incorporated in the Barcelona Clinic Liver Cancer classification, the most widely used staging system for HCC, which has been endorsed by the American and European HCC guidelines [16, 17].

### Statistics

Baseline characteristics were summarized using descriptive statistics. Chi square test or Fisher’s exact test were used to compare nominal data. Overall survival (OS) was defined as the time from date of diagnosis (date of biopsy if available or diagnostic imaging) until date of death or last contact. Survival curves were calculated using the Kaplan-Meier method and were compared by means of the log rank test (univariate analysis). Variables that reached a p-value of <0.05 in univariate analysis were entered into a multivariate analysis. The multivariate analysis was performed using a Cox proportional hazard regression model. Statistical tests were two-sided and a p-value <0.05 was considered significant. All statistical analyses were performed using SPSS version 17.0 (SPSS Inc., Chicago, IL).

## Results

### Patient characteristics

**Fig 1:**
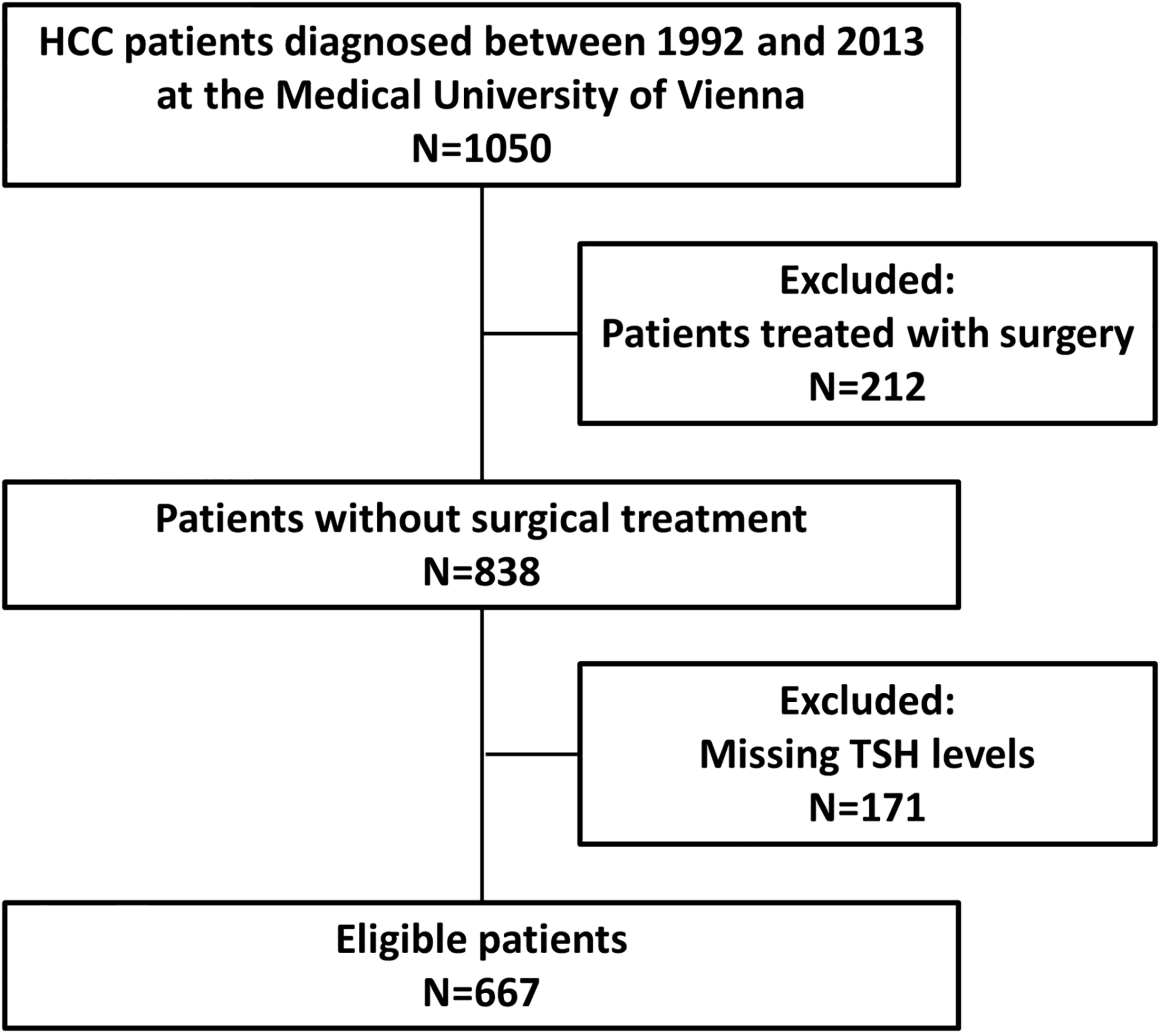
Flow chart of patient selection. Abbreviations: HCC, hepatocellular carcinoma; TSH, thyroid-stimulating hormone.

Of 667 patients included (Fig 1), 82% of the patients were male, with a male to female ratio of 4.6:1. The median age at diagnosis was 64 years (range, 32–87 years). Six percent of all patients were on thyroid hormone substitution. Detailed patient characteristics are shown in Table 1. Additional information on thyroid function is given in S1 Table. Mean follow-up was 65.5 months. Five hundred and forty-four (82%) patients died during the observation period.

**Table 1:** Patient characteristics.

		N = 667	100%
**Age (years)**	Mean±SD	64±9.6	
	Range	32–87	
**Sex**	Male	547	82
	Female	120	18
**Diabetes**	NIDDM	152	23
	IDDM	78	12
	None	437	66
**BMI (kg/m**^**2**^**)**^1^	Mean±SD	27±4.8	
**Etiology**	Alcohol	304	46
	HCV	194	29
	HBV	52	8
	NASH	20	3
	Cryptogenic	65	10
	other	32	5
**Child-Pugh**	A	286	43
	B	219	33
	C	162	24
**MELD**	Mean±SD	13±5.7	
**ECOG PS**	0	328	49
	≥1	339	51
**Largest tumor**	≤5cm	313	47
	>5cm	354	53
**Macrovascular Invasion**	No	489	73
	Yes	178	27
**Extrahepatic metastases BCLC stage**	No	577	87
	Yes	90	14
	A	90	14
	B	143	21
	C	268	40
	D	166	25
**First line therapy**	PEI/RFA	138	21
	TACE	180	27
	Sorafenib	63	9
	BSC	152	23
	Other	134	20
**CRP (mg/dl)**^2^	Mean±SD	2.62±3.59	
**AFP (IU/ml)**^3^	Mean±SD	5982±25114	
**TSH (uU/ml)**	Mean±SD	2.2±2.3	
**fT**_**4**_ **(ng/dl)**^4^	Mean±SD	1.3±0.3	
**Thyroid hormone substitution**	Yes	41	6
	No	626	94

**Abbreviations**: AFP, α-fetoprotein; BCLC, Barcelona-Clinic Liver Cancer; BMI, body mass index; BSC, best supportive care; CRP, C-reactive protein; ECOG PS, Eastern Cooperative Oncology Group performance status; fT_4_, free tetraiodthyronine; HBV, hepatitis B virus; HCV, hepatitis C virus; MELD, model of end-stage liver disease; NASH, non-alcoholic steatohepatitis; (N)IDDM, (non) insulin dependent diabetes mellitus; PEI, percutaneous ethanol injection; RFA, radiofrequency ablation; TACE, transarterial chemoembolization; TSH, thyroid-stimulating hormone.

^**1**^ missing, n = 23;

^**2**^ missing, n = 36;

^**3**^ missing, n = 9;

^**4**^ missing, n = 354

### Association of thyroid-stimulating hormone (TSH) and free tetraiodthyronine (fT_4_) with patient, liver, and tumor characteristics

TSH abnormalities significantly differed between age groups. While patients with low TSH concentrations were generally older (age >65 vs. ≤65years, 68 vs. 32%) those with high TSH (>3.77uU/ml) levels were predominantly younger (age >65 vs. ≤65years, 39 vs. 61%; p = 0.007). Thyroid hormone substitution was more often observed in patients with low TSH (<0.44uU/ml) concentration (24%) compared to those with normal (4%) or elevated TSH (13%) levels (p<0.001). Finally, patients with high TSH concentrations had larger tumors (tumor >5cm vs. ≤5cm, 61 vs. 39%) while the opposite was true for patients with low TSH concentrations (tumor >5cm vs. ≤5cm, 32 vs. 68%; p = 0.005). No association was observed between other variables including e.g. Child-Pugh stage, metastasis, or macrovascular invasion (Table 2). Notably, in patients with larger HCC (>5cm), TSH levels were only significantly associated with sex and thyroid hormone substitution, but not with other variables that might correlate with larger HCC (i.e., etiology, Child-Pugh stage) (S2 Table).

**Table 2:** Association between thyroid-stimulating hormone (TSH) and patient, liver, and tumor characteristics (n = 667).

		TSH, N (%)				
		N	low	normal	high	p-value
**Sex**	Male	547	36 (72)	458 (84)	53 (77)	
	Female	120	14 (28)	90 (16)	16 (23)	0.062
**Age**	≤65	339	16 (32)	281 (51)	42 (61)	
	>65	328	34 (68)	267 (49)	27 (39)	0.007
**Diabetes**	NIDDM	152	9 (18)	124 (23)	19 (28)	
	IDDM	78	4 (8)	69 (13)	5 (7)	
	None	437	37 (74)	355 (65)	45 (65)	0.418
**BMI (kg/m**^**2**^**)**^1^	<18.5	6	1 (2)	5 (1)	0 (0)	
	18.5–25	229	20 (40)	189 (36)	20 (29)	
	>25	409	29 (58)	332 (63)	48 (71)	0.470
**Etiology**	Alcohol	304	25 (50)	254 (46)	25 (36)	
	HCV	194	10 (20)	156 (29)	28 (41)	
	HBV	52	4 (8)	46 (8)	2 (3)	
	NASH	20	0 (0)	19 (4)	1 (1)	
	Other	97	11 (22)	73 (13)	13 (19)	0.083
**Thyroid hormone substitution**	Yes	41	12 (24)	20 (4)	9 (13)	
	No	626	38 (76)	528 (96)	60 (87)	<0.001
**Child-Pugh**	A	286	21 (42)	244 (45)	21 (30)	
	B	219	15 (30)	178 (33)	26 (38)	
	C	162	14 (28)	126 (23)	22 (32)	0.216
**MELD**	<12	361	27 (54)	302 (55)	32 (46)	
	≥12	306	23 (46)	246 (45)	37 (54)	0.390
**Largest tumor**	≤5cm	313	34 (68)	252 (46)	27 (39)	
	>5cm	354	16 (32)	296 (54)	42 (61)	0.005
**Macrovascular invasion**	No	489	41 (82)	403 (74)	45 (65)	
	Yes	178	9 (18)	145 (27)	24 (35)	0.119
**Extrahepatic metastases**	No	577	44 (88)	470 (86)	63 (91)	
	Yes	90	6 (12)	78 (14)	6 (9)	0.425
**CRP (mg/dl)**^2^	<1	285	22 (48)	239 (46)	24 (36)	
	≥1	346	24 (52)	279 (54)	43 (64)	0.260
**AFP (IU/ml)**^3^	≤100	362	23 (47)	305 (57)	34 (49)	
	>100	296	26 (53)	235 (44)	35 (51)	0.262

**Abbreviations**: AFP, α-fetoprotein; BMI, body mass index; CRP, C-reactive protein; HBV, hepatitis B virus; HCV, hepatitis C virus; MELD, model of end-stage liver disease; NASH, non-alcoholic steatohepatitis; (N)IDDM, (non) insulin dependent diabetes mellitus; TSH, thyroid-stimulating hormone.

**Definitions**: TSH low, <0.44uU/ml; TSH normal, 0.44–3.77uU/ml; TSH high, >3.77uU/ml

^**1**^ missing, n = 23;

^**2**^ missing, n = 36;

^**3**^ missing, n = 9

Free T_4_ (fT_4_) levels were available in 313 patients. Since sample size of hypothyroid patients (fT_4_ levels below 0.76ng/dl) was too small (n = 4) to allow for robust analysis, fT_4_ levels were divided into two groups (≤1.66 and >1.66ng/dl, hereafter referred to as ‘normal’ and ‘elevated’, respectively). The proportion of female patients was higher in patients with elevated fT_4_ levels (normal vs. elevated fT_4_, 16 vs. 36%; p = 0.025). Thyroid hormone substitution was more frequently observed in patients with elevated fT_4_ (normal vs. elevated fT_4_, 6 vs. 32%; p<0.001). Finally, subjects with elevated fT_4_ were more likely to have elevated CRP levels (normal vs. elevated fT_4_, 51 vs. 88%; p = 0.001). No association was observed between other variables representing liver function and tumor burden, respectively (S3 Table).

Since information on T_3_, T_4_, and fT_3_ was missing in 82–97% of patients due to the retrospective character of this analysis (S1 Table), we could not assess their association with patient, liver, and tumor characteristics.

### Uni- and multivariate analyses of prognostic factors

Median survival of the study population (n = 667) was 9.3 months (95%CI, 7.6–11.0 months).

**Fig 2:**
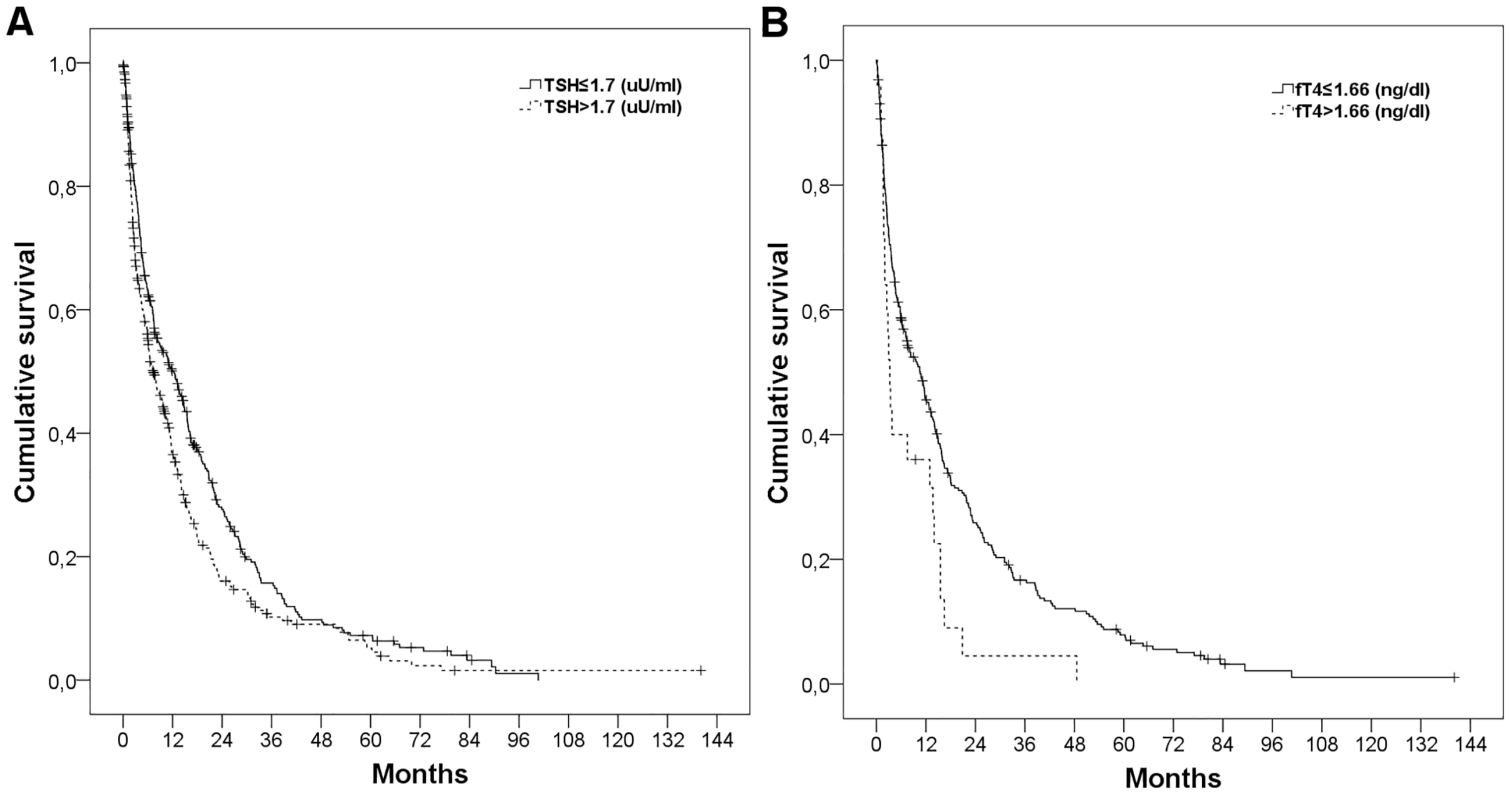
Kaplan-Meier survival curves. Overall survival (OS) according to (A) thyroid-stimulating hormone (TSH) levels (TSH≤1.7 vs. >1.7uU/ml, median OS (95%CI), 12.3 (8.9–15.7 months) vs. 7.3 months (5.4–9.2 months); p = 0.003), and (B) free tetraiodthyronine (fT4) levels (fT_4_≤1.66 vs. >1.66ng/dl, median OS (95%CI), 10.6 (7.5–13.6 months) vs. 3.3 months (2.2–4.3 months); p = 0.007).

In univariate analysis (Table 3), both, TSH (TSH≤1.7 vs. >1.7uU/ml, median OS (95%CI), 12.3 (8.9–15.7 months) vs. 7.3 months (5.4–9.2 months); p = 0.003; Fig 2A) and fT_4_ (fT_4_≤1.66 vs. >1.66ng/dl, median OS (95%CI), 10.6 (7.5–13.6 months) vs. 3.3 months (2.2–4.3 months); p = 0.007; Fig 2B) were associated with OS. Patients with manifest hyperthyroid status (n = 17) had worse survival compared to those with normal fT4 levels (i.e., euthyreosis, subclinical hyperthyreosis). The cause of death in patients with hyperthyreosis was tumor progression in 6 patients, liver failure/decompensation in 4 subjects, and unknown in 7 patients. However, thyroid status was not significantly associated with OS, most likely due to small patient numbers in some subgroups.

**Table 3:** Univariate analysis of prognostic factors (N = 667).

			Overall survival (months)		P-value
		N	Median	95% CI	(log rank)
**Age**	≤65	339	7.5	4.9–10.1	
	>65	328	10.5	8.1–12.9	0.203
**Etiology**	Viral	246	10.5	7.7–13.3	
	Others	421	8.8	6.7–10.9	0.326
**Child-Pugh**	A	286	16.2	13.8–18.7	
	B	219	7.9	5.1–10.8	
	C	162	2.4	1.9–2.9	<0.001
**Largest tumor**	≤5cm	313	14.3	11.7–16.9	
	>5cm	354	6.1	5.0–7.2	<0.001
**ECOG PS**	0	328	16.7	14.2–19.3	
	≥1	339	4.0	3.3–4.8	<0.001
**Macrovascular invasion**	No	489	12.6	10.7–14.5	
	Yes	178	3.7	2.4–5.0	<0.001
**Extrahepatic spread**	No	577	11.3	9.3–13.4	
	Yes	90	3.4	1.9–5.0	<0.001
**First-line therapy**	PEI/RFA	138	20.9	16.6–25.2	
	TACE	180	15.5	13.3–17.7	
	Sorafenib	63	8.1	4.0–12.2	
	BSC	152	1.9	1.4–2.4	
	Other	134	6.0	4.4–7.6	<0.001
**AFP (IU/ml)**^1^	≤100	362	14.0	11.8–16.2	
	>100	296	5.8	4.5–7.1	<0.001
**CRP (mg/dl)**^2^	<1	285	17.9	14.8–20.9	
	≥1	346	4.1	3.3–4.9	<0.001
**TSH (uU/ml)**	≤1.7	336	12.3	8.9–15.7	
	>1.7	331	7.3	5.4–9.2	0.003
**fT**_**4**_ **(ng/dl)**^3^	≤1.66	288	10.6	7.5–13.6	
	>1.66	25	3.3	2.2–4.3	0.007
**Thyroid hormone substitution**	Yes	41	11.9	4.0–19.7	
	No	626	9.0	7.2–10.7	0.474
**Thyroid status**	Euthyreosis	200	10.8	6.5–15.2	
	Hyperthyreosis	17	3.3	1.4–5.1	
	Subclinical hyperthyreosis	22	14.7	1.4–28.0	
	Subclinical hypothyreosis	47	6.1	0–13.5	
	Hypothyreosis	3	0.7	0.2–1.2	
	TH substitution	41	11.9	4.0–19.7	0.195

**Abbreviations**: AFP, α-fetoprotein; BSC, best supportive care; CRP, C-reactive protein; ECOG PS, Eastern Cooperative Oncology Group performance status; fT_4_, free tetraiodthyronine; MELD, model of end-stage liver disease; PEI, percutaneous ethanol injection; RFA, radiofrequency ablation; TACE, transarterial chemoembolization; TH, thyroid hormone; TSH, thyroid-stimulating hormone.

^**1**^ missing, n = 9;

^**2**^ missing, n = 36;

^3^ missing, n = 354

Other variables that were significantly associated with OS included Child-Pugh class, tumor size, Eastern Cooperative Oncology Group (ECOG) performance status (PS), macrovascular invasion (MVI), extrahepatic metastases, first-line therapy, α-fetoprotein (AFP) level, and C-reactive protein (CRP) level. Importantly, thyroid hormone substitution had no impact on OS.

Given that fT_4_ levels were only available in 313 patients, we did not include both TSH and fT_4_ in the same multivariate Cox regression model, but analyzed them separately with all other variables that were significantly associated with OS in univariate analysis. Finally, fT_4_ (HR (95%CI) for fT_4_>1.66ng/dl, 2.1 (1.3–3.3); p = 0.002) remained an independent prognostic factor for OS (Table 4), while TSH was not significantly associated with OS in multivariate analysis (S4 Table).

**Table 4:** Multivariate analysis of prognostic factors.

		Overall survival		P-value
		HR	95% CI	(Cox regression)
**Child-Pugh**	A	1		
	B	1.7	1.3–2.4	0.001
	C	2.3	1.5–3.6	<0.001
**Largest tumor**	≤5cm	1		
	>5cm	1.4	1.1–1.8	0.017
**ECOG PS**	0	1		
	≥1	1.3	0.9–1.8	0.134
**Macrovascular invasion**	No	1		
	Yes	1.2	0.9–1.7	0.285
**Extrahepatic spread**	No	1		
	Yes	1.7	1.1–2.5	0.013
**First-line therapy**	PEI/RFA	1		
	TACE	1.2	0.8–1.8	0.315
	Sorafenib	1.4	0.6–2.9	0.409
	BSC	6.0	3.8–9.4	<0.001
	Other	1.7	1.2–2.4	0.004
**AFP (IU/ml)**	≤100	1		
	>100	1.5	1.2–2.0	0.003
**CRP (mg/dl)**	<1	1		
	≥1	1.6	1.2–2.2	0.003
**fT**_**4**_ **(ng/dl)**	≤1.66	1		
	>1.66	2.1	1.3–3.3	0.002

**Abbreviations**: AFP, α-fetoprotein; BSC, best supportive care; CRP, C-reactive protein; ECOG PS, Eastern Cooperative Oncology Group performance status; fT_4_, free tetraiodthyronine; PEI, percutaneous ethanol injection; RFA, radiofrequency ablation; TACE, transarterial chemoembolization.

After separating patients into early-intermediate (BCLC A-B) and advanced-terminal (BCLC C-D) stage, fT_4_ remained associated with OS. Median survival in the early-intermediate stage group was 25.3 months (95%CI, 20.1–30.6 months) for fT_4_ ≤1.66 (n = 97) and 15.5 months (95%CI, 9.2–21.8 months) for fT_4_ >1.66 (n = 8) (p = 0.043). Similarly, in the advanced-terminal stage group, patients with fT_4_ ≤1.66 (n = 191) had a significantly (p = 0.038) longer survival of 4.5 months (95%CI, 3.1–5.8 months) compared to 2.8 months (95%CI, 1.5–4.1 months) in patients with fT_4_ >1.66 (n = 17).

## Discussion

In our cohort, 10% of patients had elevated TSH levels similar to the prevalence of hypothyroidism in patients with HCC reported by Hassan et al. (HCC patients vs. controls, 11.7 vs. 8%) [12]. Hypothyroidism has profound effects on metabolism and has therefore been linked to various conditions which either directly constitute an HCC risk factor or have the ability to contribute to the development of known predisposing conditions for HCC, such as obesity [18–21], diabetes [22–25], non-alcoholic fatty liver disease (NAFLD) [20, 26, 27], and hepatitis C infection [12, 28, 29]. Interestingly, Tarantino and colleagues reported that BMI predicted the presence of spleno-renal shunts and spleno-renal shunts were associated with an increased HCC incidence [30].

However, in our study, high serum TSH concentration was neither associated with type II diabetes mellitus nor BMI. Although etiology of liver disease was not significantly associated with TSH levels, HCV was more frequently observed in patients with high TSH levels (41%) compared to those with normal (29%) or low (20%) TSH. In contrast, Reddy and colleagues [31] found that hypothyroidism was more prevalent in patients with unknown etiology than in those with HCV or alcoholic liver disease. Additionally, we observed NASH only in patients with normal or elevated TSH but not in those with low TSH levels.

Notably, elevated TSH was associated with larger tumors in our study. In contrast, in breast cancer, hypothyroid patients were more likely to be diagnosed with a smaller tumor and at an earlier stage compared to euthyroid patients [32]. Moreover, in prostate cancer, serum T_3_ was higher in more advanced clinical stage, even though none of the men had levels above the normal range [33].

In terms of survival, patients with higher TSH levels showed a significantly worse outcome in univariate analysis. However, this effect did not hold true upon multivariate analysis when adjusting for other prognostic factors including Child-Pugh class, tumor size, performance status, macrovascular invasion, extrahepatic spread, tumor treatment, AFP, and CRP levels.

Next, we investigated the prevalence of abnormal fT_4_ levels, available in 313 of 667 patients. Elevated fT_4_ was found in 25 patients (4% of all patients, n = 667; 8% of those whose fT_4_ levels where available, n = 313). In comparison, a large study conducted in the United States reported a prevalence of only 0.5% for clinical hyperthyroidism. Notably, they used total instead of free T_4_ concentrations but adjusted for the common T_4_-confounders pregnancy and estrogen therapy [34]. In an epidemiologic study by Hassan et al., the prevalence of hyperthyroidism was 1.9% among HCC patients and 1.3% among controls [12].

In our cohort, elevated fT_4_ concentrations were not associated with etiology of liver disease and variables representing liver function or tumor burden. However, patients with elevated fT_4_ more frequently had elevated CRP levels which indicate worse prognosis in HCC and could be a reflection of the “inflammatory field effect” [35]. This effect could directly fuel tumor progression as CRP levels are an accepted surrogate marker for the release of Interleukin (IL) -6, an important regulator of CRP secretion, which in turn is associated with both acceleration of HCC development and metastasis [35–38]. Additionally, thyroid hormone replacement was more common in patients with increased fT_4_ levels (fT_4_≤1.66 vs. >1.66, 6 vs. 32%).

We next investigated the impact of fT_4_ on the prognosis of HCC patients and found that elevated fT_4_ levels at the time of HCC diagnosis were significantly associated with poor OS. This effect held true even after adjusting for other known prognostic factors for HCC in a multivariate Cox regression model.

These results are supported by several preclinical studies showing that thyroid hormone signaling promotes tumor invasiveness and metastasis [2, 13, 15, 39, 40], and could be of clinical relevance mainly for two reasons. First, fT_4_ is a valuable prognostic parameter in HCC that is widely available, non-invasively collectable, and objective. Second, considering both the association between thyroid hormone substitution and elevated fT_4_ levels as well as the strong prognostic relevance of elevated fT_4_, our data could suggest some caution when replacing thyroid hormones in patients with established HCC, as hormone replacement-induced fT_4_ elevation might render tumors more aggressive. This should especially be considered in patients with advanced HCC receiving sorafenib therapy where hypothyroidism often occurs [41–43], and might prompt physicians to initiate hormone replacement therapy.

Notably, other studies reported that T_3_ treatment led to regression of preneoplastic lesions in rodent models of heptocarcinogenesis [44, 45].

The main limitation of this study was the lack of patients with low fT_4_ levels, which precluded the investigation of effects of overt hypothyroidism on HCC and only allowed analyzing the effect of hypothyroidism using TSH levels. This especially impeded the analysis of the impact of thyroid hormones on HCC with metabolic background. Furthermore, the small sample size of patients with abnormal fT_4_ levels did not allow subgroup analyses within the BCLC stages and Child-Pugh class. As thyroid hormones may exert opposing effects at different stages of HCC development and progression some effects could evade detection when analysis is performed without differentiation according to tumor stage [2]. However, after grouping patients into early-intermediate (BCLC A-B) and advanced-terminal stage (BCLC C-D), the negative impact of elevated fT_4_ on survival remained significant in both subgroups. Additionally, non-thyroidal illness syndrome (NTIS), characterized by low serum T_3_ with normal T_4_ levels, is associated with HCC and other malignancies [46, 47]. The fact that T_3_ and fT_3_ levels were missing in most of our patients represents another potential bias we could not address in our analysis. In light of the stated limitations, this study could not adequately investigate the hypothesis of a dual role of thyroid hormones in HCC [2].

In conclusion, high TSH level was associated with larger tumor size but not with survival when adjusted for known prognostic factors for HCC. Elevation of fT_4_ resulted in poor survival and remained an independent prognostic factor for OS. These results can be considered as hypothesis-generating paving the path for further work. Prospective studies including clinically hypothyroid patients and subgroup analyses of all tumor stages are needed to further elucidate the role of thyroid hormones in HCC.

## Supporting information

S1 Table Thyroid function. (DOCX)

S2 Table Association between TSH and patient, liver, and tumor characteristics in patients with the largest tumor being >5cm. (DOCX)

S3 Table Association between free tetraiodthyronine (fT_4_) and patient, liver, and tumor characteristics (n = 313). (DOCX)

S4 Table Multivariate analysis of prognostic factors. (DOCX)
