# Supplementary material for: The impact of thyroid hormones on patients with hepatocellular carcinoma
Source: PLoS One. 2017 Aug 3;12(8):e0181878. doi: 10.1371/journal.pone.0181878 (PMC5542594; doi:10.1371/journal.pone.0181878)
Supplement: S1 Table — (DOCX) [file pone.0181878.s001.docx]

**S1 Table. Thyroid function.**

|  |  | **N=667** | **100%** |
| --- | --- | --- | --- |
| **TSH (uU/ml)** | <0.44 | 50 | 8 |
|  | 0.44-3.77 | 548 | 82 |
|  | >3.77 | 69 | 10 |
| **T_3_ (ng/ml)** | <0.8 | 15 | 2 |
|  | 0.8-1.8 | 23 | 3 |
|  | >1.8 | 1 | 0.1 |
|  | Missing | 628 | 94 |
| **T_4_ (ng/ml)** | <58 | 6 | 1 |
|  | 58-124 | 87 | 13 |
|  | >124 | 26 | 4 |
|  | Missing | 548 | 82 |
| **fT_3_ (pg/ml)** | <2.15 | 4 | 1 |
|  | 2.15-4.12 | 13 | 2 |
|  | >4.12 | 2 | 0.3 |
|  | Missing | 648 | 97 |
| **fT_4_ (ng/dl)** | <0.76 | 4 | 1 |
|  | 0.76-1.66 | 284 | 43 |
|  | >1.66 | 25 | 4 |
|  | Missing | 354 | 53 |
| **Inderal therapy** | Yes | 98 | 15 |
|  | No | 569 | 85 |
|  | | | |

**Abbreviations:** (f)T_3_, (free) triiodthyronine; (f)T_4_, (free) tetraiodthyronine; TSH, thyroid stimulating hormone.
