# Supplementary material for: The impact of thyroid hormones on patients with hepatocellular carcinoma
Source: PLoS One. 2017 Aug 3;12(8):e0181878. doi: 10.1371/journal.pone.0181878 (PMC5542594; doi:10.1371/journal.pone.0181878)
Supplement: S2 Table — (DOCX) [file pone.0181878.s002.docx]

| **S2 Table.** **Association between TSH and patient, liver, and tumor characteristics in patients with the largest tumor being >5cm** | | | | | | |
| --- | --- | --- | --- | --- | --- | --- |
|  |  |  |  | | **TSH, N (%)** |  |
|  |  | **N** | **low** | **normal** | **high** | |
| **Sex** | Male | 297 | 12 (75) | 255 (86) | 30 (71) | |
|  | Female | 57 | 4 (25) | 41 (14) | 12 (29) | |
| **Age** | ≤65 | 179 | 4 (25) | 150 (51) | 25 (60) | |
|  | >65 | 175 | 12 (75) | 146 (49) | 17 (41) | |
| **Diabetes** | NIDDM | 86 | 4 (25) | 69 (23) | 13 (31) | |
|  | IDDM | 34 | 1 (6) | 31 (11) | 2(5) | |
|  | None | 234 | 11 (69) | 196 (66) | 27 (64) | |
| **BMI (kg/m^2^)^1^** | <18.5 | 2 | 0 (0) | 2 (1) | 0 (0) | |
|  | 18.5-25 | 123 | 6 (38) | 104 (36) | 13 (31) | |
|  | >25 | 219 | 10 (63) | 180 (63) | 29 (69) | |
| **Etiology** | Alcohol | 156 | 8 (50) | 134 (45) | 14 (33) | |
|  | HCV | 96 | 2 (13) | 80 (27) | 14 (33) | |
|  | HBV | 27 | 2 (13) | 23 (8) | 2 (5) | |
|  | NASH | 10 | 0 (0) | 10 (3) | 0 (0) | |
|  | Other | 65 | 4 (25) | 49 (17) | 12 (29) | |
| **Thyroid hormone** | Yes | 24 | 4 (25) | 14 (5) | 6 (14) | |
| **substitution** | No | 330 | 12 (75) | 282 (95) | 36 (86) | |
| **Child-Pugh** | A | 160 | 11 (69) | 136 (46) | 13 (31) | |
|  | B | 111 | 2 (13) | 91 (31) | 18 (43) | |
|  | C | 83 | 3 (19) | 69 (23) | 11 (26) | |
| **MELD** | <12 | 185 | 10 (63) | 153 (52) | 22 (52) | |
|  | ≥12 | 169 | 6 (38) | 143 (48) | 20 (48) | |
| **Macrovascular** | No | 216 | 12 (75) | 181 (61) | 23 (55) | |
| **invasion** | Yes | 138 | 4 (25) | 115 (39) | 19 (45) | |
| **Extrahepatic** | No | 296 | 15 (94) | 244 (82) | 37 (88) | |
| **metastases** | Yes | 58 | 1 (6) | 52 (18) | 5 (12) | |
| **CRP (mg/dl)^2^** | <1 | 116 | 8 (53) | 97 (34) | 11 (27) | |
|  | ≥1 | 222 | 7 (47) | 185 (66) | 30 (73) | |
| **AFP (IU/ml)^3^** | ≤100 | 164 | 8 (53) | 140 (48) | 16 (38) | |
|  | >100 | 187 | 7 (47) | 154 (52) | 26 (62) | |
| **Abbreviations:** AFP, α-fetoprotein; BMI, body mass index; CRP, C-reactive protein; HBV, hepatitis B virus; HCV, hepatitis C virus; MELD, model of end-stage liver disease; NASH, non-alcoholic steatohepatitis; (N)IDDM, (non) insulin dependent diabetes mellitus; TSH, thyroid-stimulating hormone.  **Definitions:** TSH low, <0.44uU/ml; TSH normal, 0.44-3.77uU/ml; TSH high, >3.77uU/ml  **^1^** missing, n=10; **^2^** missing, n=16; **^3^** missing, n=3 | | | | | | |
