# Supplementary material for: The impact of thyroid hormones on patients with hepatocellular carcinoma
Source: PLoS One. 2017 Aug 3;12(8):e0181878. doi: 10.1371/journal.pone.0181878 (PMC5542594; doi:10.1371/journal.pone.0181878)
Supplement: S3 Table — (DOCX) [file pone.0181878.s003.docx]

| **S3 Table. Association between free tetraiodthyronine (fT_4_) and patient, liver, and tumor characteristics (n=313).** |
| --- |

|  |  |  | **fT_4_ (ng/dl), N (%)** | |  |  |
| --- | --- | --- | --- | --- | --- | --- |
|  |  | **N** | **≤1.66** | **>1.66** | **p-value** | |
| **Sex** | Male | 257 | 241 (84) | 16 (64) |  | |
|  | Female | 56 | 47 (16) | 9 (36) | 0.025 | |
| **Age** | <65 | 155 | 146 (51) | 9 (36) |  | |
|  | ≥65 | 158 | 142 (49) | 16 (64) | 0.159 | |
| **Diabetes** | NIDDM | 74 | 70 (24) | 4 (16) |  | |
|  | IDDM | 35 | 31 (11) | 4 (16) |  | |
|  | None | 204 | 187 (65) | 17 (68) | 0.511 | |
| **BMI (kg/m^2^)^1^** | <18.5 | 2 | 2 (1) | 0 (0) |  | |
|  | 18.5-25 | 120 | 106 (38) | 14 (56) |  | |
|  | >25 | 181 | 170 (61) | 11 (44) | 0.236 | |
| **Etiology** | Alcohol | 151 | 139 (48) | 12 (48) |  | |
|  | HCV | 94 | 87 (30) | 7 (28) |  | |
|  | HBV | 21 | 20 (7) | 1 (4) |  | |
|  | NASH | 1 | 1 (0.3) | 0 (0) |  | |
|  | Other | 46 | 41 (14) | 5 (20) | 0.879 | |
| **Thyroid hormone** | Yes | 24 | 16 (6) | 8 (32) |  | |
| **substitution** | No | 289 | 272 (94) | 17 (68) | <0.001 | |
| **Child-Pugh** | A | 127 | 119 (41) | 8 (32) |  | |
|  | B | 100 | 92 (32) | 8 (32) |  | |
|  | C | 86 | 77 (27) | 9 (36) | 0.546 | |
| **MELD** | <12 | 168 | 159 (55) | 9 (36) |  | |
|  | ≥12 | 145 | 129 (45) | 16 (64) | 0.065 | |
| **Largest tumor** | ≤5cm | 163 | 150 (52) | 13 (52) |  | |
|  | >5cm | 150 | 138 (48) | 12 (48) | 0.994 | |
| **Macrovascular** | No | 238 | 219 (76) | 19 (76) |  | |
| **invasion** | Yes | 75 | 69 (24) | 6 (24) | 0.996 | |
| **Extrahepatic** | No | 279 | 259 (90) | 20 (80) |  | |
| **metastases** | Yes | 34 | 29 (10) | 5 (20) | 0.169 | |
| **CRP (mg/dl)^2^** | <1 | 138 | 135 (49) | 3 (13) |  | |
|  | ≥1 | 160 | 139 (51) | 21 (88) | 0.001 | |
| **AFP (IU/ml)^3^** | ≤100 | 174 | 160 (56) | 14 (58) |  | |
|  | >100 | 136 | 126 (44) | 10 (42) | 0.821 | |

**Abbreviations:** AFP, α-fetoprotein; BMI, body mass index; CRP, C-reactive protein; HBV, hepatitis B virus; HCV, hepatitis C virus; MELD, model of end-stage liver disease; NASH, non-alcoholic steatohepatitis; (N)IDDM, (non) insulin dependent diabetes mellitus; fT_4_, free tetraiodthyronine.

**^1^** missing, n=10; **^2^** missing, n=15; **^3^** missing, n=3
