# Supplementary material for: The impact of thyroid hormones on patients with hepatocellular carcinoma
Source: PLoS One. 2017 Aug 3;12(8):e0181878. doi: 10.1371/journal.pone.0181878 (PMC5542594; doi:10.1371/journal.pone.0181878)
Supplement: S4 Table — (DOCX) [file pone.0181878.s004.docx]

**S 4 Table. Multivariate analysis of prognostic factors.**

|  |  | **Overall survival** | | **P-value** |
| --- | --- | --- | --- | --- |
|  |  | **HR** | **95% CI** | **(Cox regression)** |
| **Child-Pugh** | A | 1 |  |  |
|  | B | 1.6 | 1.3-2.0 | <0.001 |
|  | C | 2.2 | 1.6-2.9 | <0.001 |
| **Largest tumor** | ≤5cm | 1 |  |  |
|  | >5cm | 1.4 | 1.1-1.6 | 0.002 |
| **ECOG PS** | 0 | 1 |  |  |
|  | ≥1 | 1.4 | 1.1-1.7 | 0.009 |
| **Macrovascular** | No | 1 |  |  |
| **invasion** | Yes | 1.3 | 1.1-1.7 | 0.010 |
| **Extrahepatic spread** | No | 1 |  |  |
|  | Yes | 1.4 | 1.1-1.8 | 0.013 |
| **First-line therapy** | PEI/RFA | 1 |  |  |
|  | TACE | 1.3 | 1.0-1.7 | 0.106 |
|  | Sorafenib | 1.0 | 0.7-1.6 | 0.869 |
|  | BSC | 3.2 | 2.4-4.3 | <0.001 |
|  | Other | 1.8 | 1.3-2.4 | <0.001 |
| **AFP (IU/ml)** | ≤100 | 1 |  |  |
|  | >100 | 1.3 | 1.0-1.5 | 0.018 |
| **CRP (mg/dl)** | <1 | 1 |  |  |
|  | ≥1 | 1.7 | 1.4-2.1 | <0.001 |
| **TSH (uU/ml)** | ≤1.7 | 1 |  |  |
|  | >1.7 | 1.1 | 0.9-1.4 | 0.180 |

**Abbreviations:** AFP, α-fetoprotein; BSC, best supportive care; CRP, C-reactive protein; ECOG PS, Eastern Cooperative Oncology Group performance status; PEI, percutaneous ethanol injection; RFA, radiofrequency ablation; TACE, transarterial chemoembolization; TSH, thyroid-stimulating hormone.
